# Uterine Catarrh, Frequently the Cause of Sterility

**Published:** 1871-06

**Authors:** 


Uterine Catarrh, frequently the cause of Sterility New Treatment by H. E. Gantillon, M.D. J. Campbell, publisher, 18 Tremont Street, Boston.

